# P-2088. Work Absenteeism Among Women Non-Healthcare Essential Workers During the Late-Phases of the COVID-19 Pandemic

**DOI:** 10.1093/ofid/ofaf695.2252

**Published:** 2026-01-11

**Authors:** Kimberly C Okafor, Keipp Talbot, Velma McBride Murry, Carlos G Grijalva, Bryan Blette

**Affiliations:** Vanderbilt University Medical Center, Nashville, TN; Vanderbilt University Medical Center, Nashville, Tennessee; Vanderbilt University Medical Center, Nashville, Tennessee; Vanderbilt University Medical Center, Nashville, Tennessee; Vanderbilt University Medical Center, Nashville, Tennessee

## Abstract

**Background:**

The COVID-19 pandemic worsened health and economic challenges for women essential workers. Due to their concentration in the service sectors, gender pay gaps, and limited benefits, many lacked financial security against illness or caregiving disruptions. Childcare "deserts," intensified by the pandemic, disproportionately burdened women, as they assumed additional caregiving responsibilities, contributing to destabilized employment and delayed recovery relative to men. Data on total and childcare-related absenteeism during the pandemic remains limited but is critical for informing equitable public health policy.

**Table 1:**
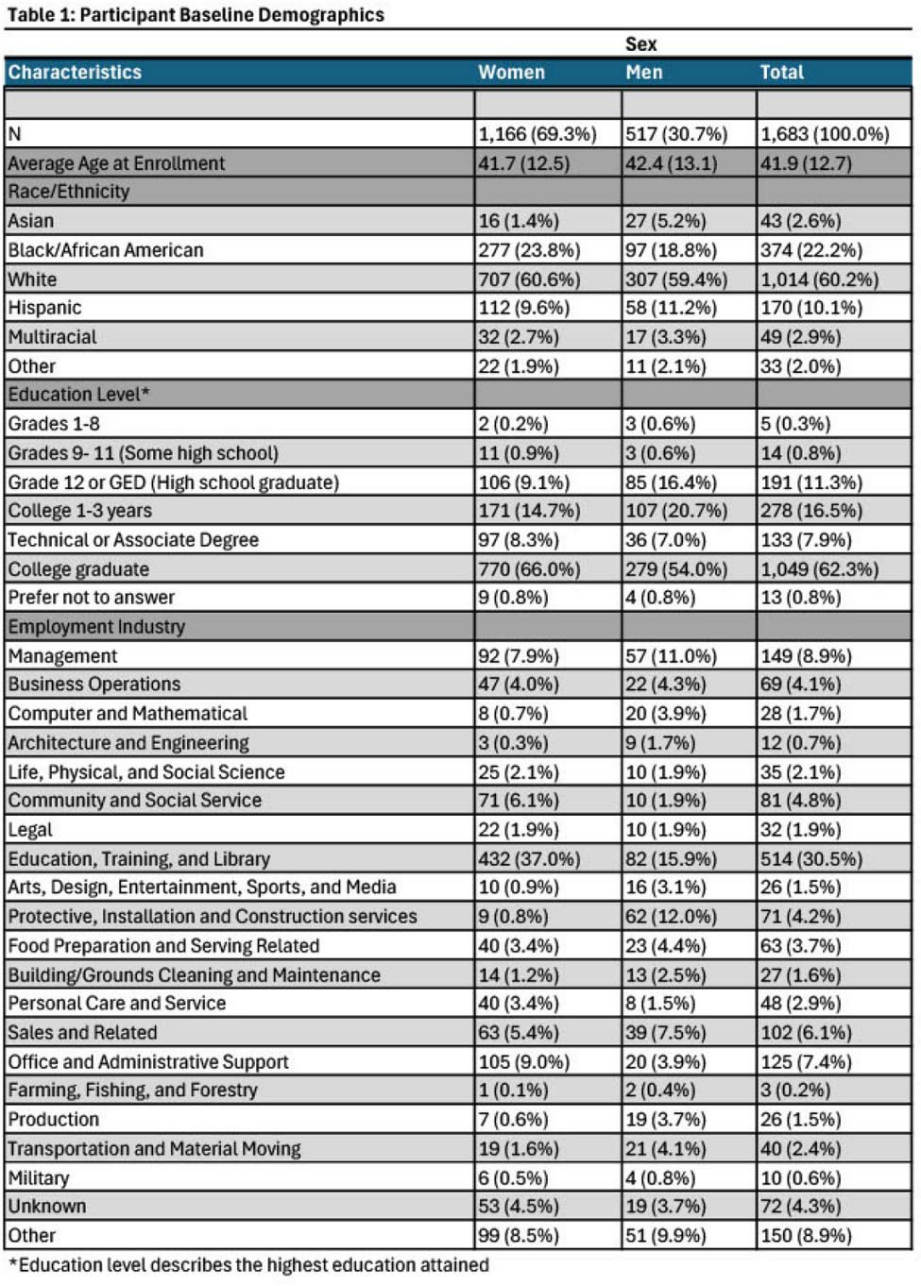
Participant Baseline Demographics

**Table 2:**
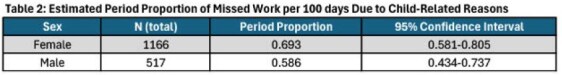
Estimated Period Proportion of Missed Work per 100 days Due to Child-Related Reasons

**Methods:**

This is a retrospective secondary analysis from the Virus and Infections in Essential Workers (VIEW) Study (2022–2024), a prospective cohort. From Middle Tennessee, 1,996 non-healthcare essential workers and household contacts were enrolled. Quarterly surveys recorded missed workdays for the month prior. Total missed days were directly reported, while child-related absences were recorded in ranges (0, 1-2, 3-6, and 7+ days). Therefore, medians of these ranges were used to estimate missed work. For 1-2, 1.5 was used, 3-6 was 4.5, and 7+ days was 8.5 days. The proportion of missed workdays per 100 days was estimated and compared between groups using multivariable Poisson regression with robust standard errors. Average child-related work absences per 30 days were calculated by summing missed workdays, divided by follow-up time (in months), and then the means among enrollees with varying numbers of household children were compared.

**Table 3:**
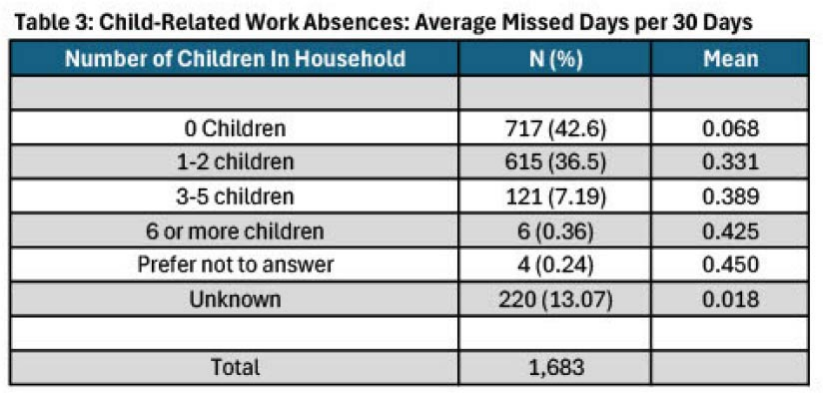
Child-Related Work Absences: Average Missed Days per 30 Days

**Results:**

The analysis included 1,683 participants who completed at least one survey; 69% were women. Descriptive statistics are listed in Table 1. Women missed 2.3 workdays per 100 days (95% CI: 2.02–2.57), whereas men missed 1.83 per 100 days (95% CI: 1.48–2.17). Child-related work absences (per 100 days) were also higher in women, although confidence intervals were wide (Table 2). Average days missed due to child-related absences (per 30 days) increased with the number of children in the home (Table 3).

**Conclusion:**

Women essential workers experienced greater total and child-related work absences than men, underscoring the need for supportive policies for this vital component of the essential workforce.

**Disclosures:**

Carlos G. Grijalva, MD MPH, AHRQ: Grant/Research Support|CDC: Grant/Research Support|GSK: Advisor/Consultant|Merck: Advisor/Consultant|NIH: Grant/Research Support|Syneos Health: Grant/Research Support

